# Alcohol-Related Disparities Among Women: Evidence and Potential Explanations

**DOI:** 10.35946/arcr.v40.2.09

**Published:** 2020-09-03

**Authors:** Nina Mulia, Kara M. Bensley

**Affiliations:** Alcohol Research Group, Public Health Institute, Emeryville, California

**Keywords:** alcohol problems, health disparities, minorities, cumulative disadvantage, life course, alcohol

## Abstract

Although research on alcohol-related disparities among women is a highly understudied area, evidence shows that racial/ethnic minority women, sexual minority women, and women of low socioeconomic status (based on education, income, or residence in disadvantaged neighborhoods) are more likely to experience alcohol-related problems. These problems include alcohol use disorder, particularly after young adulthood, and certain alcohol-related health, morbidity, and mortality outcomes. In some cases, disparities may reflect differences in alcohol consumption, but in other cases such disparities appear to occur despite similar and possibly lower levels of consumption among the affected groups. To understand alcohol-related disparities among women, several factors should be considered. These include age; the duration of heavy drinking over the life course; the widening disparity in cumulative socioeconomic disadvantage and health in middle adulthood; social status; sociocultural context; genetic factors that affect alcohol metabolism; and access to and quality of alcohol treatment services and health care. To inform the development of interventions that might mitigate disparities among women, research is needed to identify the factors and mechanisms that contribute most to a group’s elevated risk for a given alcohol-related problem.

## INTRODUCTION

Although women consume less alcohol and drink less often than men,^1^ women’s drinking warrants serious attention from alcohol researchers and health care providers, in part because women are more susceptible to certain alcohol-related problems at a given level of consumption^2^ and because women are less likely to receive help for problems with alcohol use.^3^ While women may share many experiences and risk factors relevant to their alcohol use and associated problems, women are not a monolithic group. Multiple dimensions of social location (e.g., race/ethnicity, socioeconomic status, and sexual identity) profoundly shape women’s lived experiences.^4^ These can affect health and a wide range of health-related factors over the life course, such as social and environmental risk and health-promoting exposures, health behavior, resources that enhance health and help to manage disease, care-seeking, and the quality of health care received. Thus, unsurprisingly, among women there is heterogeneity of risk for problems related to drinking.

This article briefly reviews what is known about alcohol-related disparities among women and discusses mechanisms that could give rise to inequities in alcohol outcomes. In this article, disparity refers to social group differences in which groups that have greater social or economic advantages have more desirable health outcomes than groups without those advantages.^5^ Research on alcohol-related disparities has focused on racial/ethnic and socioeconomic groups^6^–^8^ and often has not been stratified by gender to examine disparities among women or men separately, as doing so would require very large samples for low-prevalence outcomes. Thus, this review reflects a predominant focus in the extant literature on race/ethnicity (often White, Black, and Latinx groups, with rare analysis of Latinx subgroups), socioeconomic status, and the limited study of disparities among women. Far less research has been conducted on sexual minority groups (defined by sexual orientation). Reflecting the work to date, unless otherwise stated, this review defines women based on physiological sex. Finally, this review focuses on problems associated with personal alcohol consumption and does not include the many secondary harms experienced because of other people’s drinking.

## DISPARITIES IN ALCOHOL-RELATED PROBLEMS

Identifying racial/ethnic and socioeconomic disparities in alcohol-related problems is not always a straightforward task, partly because of differential abstinence rates across racial/ethnic and socioeconomic groups. For example, in the National Epidemiologic Survey on Alcohol and Related Conditions-III (NESARC-III), the percentage of people who drank alcohol in the past year ranged from 62% to 75% across racial/ethnic groups and 56% to 81% across levels of education.^1^ The National Alcohol Survey (NAS) reported 64% of heterosexual women and 78% of bisexual women drank alcohol in the past year.^9^ In addition, race, ethnicity, and socioeconomic status are deeply intertwined in the United States.^10^ In light of the above, the detection of alcohol-related disparities can be affected by the inclusion of abstainers in analyses and also by how investigators handle socioeconomic status when analyzing racial/ethnic differences. Although analytic decisions depend on research objectives (e.g., to establish general population rates, understand risk relationships, estimate residual racial/ethnic differences, or recognize the role of socioeconomic status in racial/ethnic differences), sensitivity analyses are always a useful option to gauge the effects of such decisions on study results and enhance interpretation. Effort was made in this review to be attentive to such decisions.

### Alcohol Use Disorder and Negative Consequences of Drinking

The following section provides a review of research on the prevalence and risk of alcohol-related problems in different subgroups of women defined by race/ethnicity, socioeconomic status, and sexual minority status. Problems examined in this literature include alcohol use disorder (AUD) and negative consequences of drinking. In nearly all of the studies reviewed, AUD was defined according to the *Diagnostic and Statistical Manual of Mental Disorders, Fourth Edition (DSM-IV),*^11^ which includes and distinguishes alcohol abuse and alcohol dependence. In 2013, the *Diagnostic and Statistical Manual of Mental Disorders, Fifth Edition (DSM-5)*^12^ was released, which replaces DSM-IV alcohol abuse and dependence diagnoses with a single AUD diagnosis that is classified as mild, moderate, and severe.

#### Race and ethnicity

National survey data show greater prevalence of DSM-IV AUD among White women compared to other racial/ethnic groups. For example, in Wave 1 of the NESARC, which was conducted from 2001 to 2002, age group–specific rates of DSM-IV alcohol abuse and dependence among women (including abstainers) were consistently higher in White women compared to Black, Latina, and Asian/Pacific Islander women in nearly all of four age groups examined.^13^ The exceptions were American Indian/Alaska Native (AIAN) women, whose prevalence of DSM-IV alcohol abuse and dependence was greater than that of White women in three of four age groups, and Black women, whose DSM-IV alcohol dependence prevalence was higher than that of White women at midlife (ages 45 to 64) and older (ages 65 and older). However, many of these differences did not appear to be statistically significant. Taking into account standard error, the clearest differences were observed among White, Black, and Latina women, the three largest groups. DSM-IV alcohol abuse prevalence was higher in White women compared to Black women before midlife (younger than age 45), and higher than DSM-IV alcohol abuse prevalence of Latinas in all but the oldest age group (ages 65 and older).

In the same NESARC survey, the prevalence of DSM-IV alcohol dependence was significantly higher only in young-adult, White women (ages 18 to 29) at 6% vs. 4% in young Black women and 4% in young Latina women.^13^ At 9%, the prevalence of DSM-IV alcohol dependence among young AIAN women was highest of all, but it had a wide confidence interval. By contrast, in 2000, 2005, and 2010 NAS data, White, Black, and Latina women (including abstainers and not stratified by age) showed statistically nondistinguishable prevalence and odds of having DSM-IV alcohol dependence and two or more negative consequences of drinking.^14^

Because these studies were based on older data that, in some cases, were collected nearly 20 years ago, data from the 2017 National Survey on Drug Use and Health (NSDUH)^15^ were analyzed to provide updated national estimates for women. As shown in Table 1, most of the significant racial/ethnic differences in DSM-IV alcohol dependence prevalence were no longer apparent when abstainers were excluded. When compared with White women who drink alcohol, only Asian women who drink had significantly lower rates of DSM-IV AUD, and AIAN women who drink had higher rates of DSM-IV AUD.

In studies excluding lifetime abstainers, there is some evidence of greater alcohol problems among racial/ethnic minority women who drink compared with White women who drink. For example, Grant and colleagues conducted a longitudinal analysis of NESARC Waves 1 and 2 from the early 2000s and found that at Wave 2, young White women had the greatest risk for DSM-IV alcohol dependence onset compared with young Black and Latina women.^16^ However, the risk for young White women was lower than that for older minority women. Both Black and U.S.-born Latina women ages 40 and older had greater risk of DSM-IV alcohol dependence onset than young White women (adjusted *OR* = 1.71 and 2.08, respectively).^16^ In addition, older Black and U.S.-born Latina women had more persistent alcohol dependence (adjusted *OR* = 2.73 and 1.36, respectively), and older U.S.-born Latina women had greater recurrence of dependence (among those with lifetime dependence prior to Wave 1). This elevated risk among older minority women was in marked contrast to similarly aged, White peers, whose risk for alcohol dependence onset, persistence, and recurrence was much lower than that of young White women. The racial/ethnic patterning of risk was the same when DSM-IV AUD was the outcome, except that disparities were also evident among younger minority women ages 30 to 39. In this age group, Black women had greater AUD onset, and U.S.-born Latinas had greater AUD persistence than young White women.

Notably, this NESARC study did not control for socioeconomic status indicators.^16^ In a 2005 and 2010 combined NAS study of women who drink, which adjusted for demographics, education, and income and also rigorously controlled for heavy drinking, the only disparities found between Black and White women were in DSM-IV alcohol dependence (adjusted *OR* = 3.3), and this disparity held across the range of heavy drinking.^17^ There was no significant disparity between Latina and White women in either negative consequences of drinking (an outcome similar to alcohol abuse) or DSM-IV alcohol dependence. (Due to sample size limitations of the study,^17^ U.S.-born Latina women were not analyzed separately as they were in the NESARC study by Grant and colleagues.^16^)

As noted, all of the research on AUD in demographic subgroups reviewed above, including the 2017 NSDUH data on AUD,^15^ is based on the DSM-IV diagnostic criteria rather than the DSM-5 criteria. Thus, it is not clear whether these findings (especially those based on data collected from the early 2000s) accurately reflect DSM-5 AUD patterns among women, as the latter have not yet been examined. However, results from two recent NESARC-III studies of women and men combined suggest that the patterning of AUD prevalence across racial/ethnic, socioeconomic, and other demographic subgroups may be similar across DSM-IV and DSM-5 criteria.^18^,^19^ For instance, AUD prevalence among White, Black, and Latinx study participants based on DSM-IV criteria was 13%, 13%, and 12%, respectively,^18^ and the prevalence based on DSM-5 criteria was 14%, 14%, and 14%, respectively.^19^ Similarly, for educational levels, the DSM-IV AUD prevalence was 10% for less than high school, 13% for high school, and 13% for some college or more,^18^ and the prevalence based on DSM-5 criteria was 12%, 15%, and 14%, respectively.^19^ These results suggest that the presence or absence of disparities in women’s prevalence of DSM-5 AUD might reasonably be gauged by recent research that uses DSM-IV AUD criteria (for instance, as captured by the 2017 NSDUH). But confirmation is needed, as the NESARC-III analyses were not restricted to women.

#### Socioeconomic status

Similar to the findings for race/ethnicity, the 2017 NSDUH data show significant differences in DSM-IV alcohol dependence and AUD by educational attainment, but when abstainers are excluded, nearly all differences become nonsignificant (see Table 1).^15^ Importantly, in a recent systematic review, Collins concluded that although groups with greater socioeconomic advantages (defined by income, education, and other indicators at the individual, family, or neighborhood levels) had similar or greater levels of alcohol consumption than those with fewer advantages, the groups with fewer socioeconomic advantages were at greater risk for alcohol-related problems.^8^ This finding has been referred to as the “alcohol harm paradox”^20^ and is similar to the phenomenon among some U.S. racial/ethnic minority groups, particularly Black persons, of having greater risk for alcohol-related problems than White persons despite drinking less.^21^

This socioeconomic status paradox has been studied mostly outside of the United States and has been observed for a variety of alcohol outcomes. A meta-analysis by Grittner and colleagues, drawing upon survey data from 25 countries, found that in several high-income countries, women who drink alcohol and who have less education were at greater risk for external drinking consequences (e.g., consequences affecting finances; work, school, or employment; close relationships; and risk of injury/fights).^22^ In the full sample of countries, an inverse educational gradient was found when controlling for age and drinking pattern, as well as country-level, socioeconomic development factors.

The socioeconomic conditions of residential neighborhoods also are relevant. Analysis of the 2000 and 2005 combined NAS data found that women who drink alcohol and live in disadvantaged neighborhoods have twofold greater risk for alcohol problems (adjusted *OR* = 2.07 for two or more drinking consequences or DSM-IV alcohol dependence) than women who drink and live in more advantaged neighborhoods.^23^ This study controlled for individuals’ education, income, unemployment status, and demographics.

A different study that used 2000 and 2005 combined NAS data further showed that among White women who drink alcohol, neighborhood disadvantage was associated with increased risk for negative consequences of drinking.^24^ The authors noted that White women who drink and reside in disadvantaged (as compared to more advantaged) neighborhoods were challenged by greater family histories of alcohol problems, co-occurring drug use, and drinking to cope with stress, which are risk factors for alcohol problems.

Providing a context for such findings, a longitudinal study of women in poverty highlighted the distinctive stressors faced by women who drink and have low incomes.^25^ Stressful life events and neighborhood stressors (e.g., crime, drug trafficking, and shootings) were common, and these in addition to economic stress, contributed to psychological distress and increased women’s risk for developing problematic alcohol use.

#### Sexual minority women

In this article, sexual minority women, including bisexual women and lesbians, are defined based on sexual orientation. In a study by Wilsnack and colleagues, the investigators compared data collected from sexual minority women in the 2001 to 2002 Chicago Study of Health and Life Experience of Women (CHLEW) study with data collected from exclusively heterosexual women in the 2001 National Study of Health and Life Experiences of Women.^26^ The investigators found higher prevalence of lifetime alcohol-related problems, alcohol dependence symptoms, and hazardous drinking among sexual minority women. Bisexual women were most likely to report alcohol problems, with 70% reporting lifetime problems in contrast to 29% of heterosexual women.

Similar disparities in hazardous drinking were found in a more recent wave of the CHLEW study (2010 to 2012) and in a 2000 to 2015 NAS analysis.^9^ Additionally, a separate study by Drabble and colleagues that used 2000 NAS data found that lesbians had 7.1 times higher risk of meeting criteria for DSM-IV alcohol dependence (bisexual women had 6.4 times higher risk) than heterosexual women.^27^ A recent study that used 2015 to 2017 NSDUH data indicated disparities in DSM-IV AUD rates as well.^28^ In that study, bisexual women had 2.2 times higher odds than heterosexual women and 1.5 times higher odds than lesbian women of having past-year AUD after adjusting for demographic characteristics.^28^

Although this review focuses on sexual minority women, the newly emerging literature on alcohol use among gender minority women (i.e., noncisgender and nonbinary women) should be noted. A systematic review of transgender individuals (including gender minority women) by Gilbert and colleagues found estimates of binge drinking among transgender individuals ranging from 7% to 65%, with estimates of lifetime and past-year DSM-IV AUD prevalence at 26% and 11%, respectively.^29^ More research is needed on these groups. As noted by Gilbert and colleagues, to facilitate research on alcohol use disparities among gender minority women and transgender individuals, new methods will be needed, as many of the current alcohol use measures to assess unsafe drinking rely on physiological sex-specific cut points.

### Health, Morbidity, and Mortality

Disparities in alcohol-related health outcomes, morbidity, and mortality are studied less commonly than disparities in AUD and the negative consequences of drinking alcohol. Few studies focus on women; instead, studies typically include women and men and control for gender. Nonetheless, in analyses restricted to women, racial/ethnic and socioeconomic disparities in risk have been reported for some alcohol-related health conditions and outcomes. For example, based on suicide decedent data from the National Violent Death Reporting System, AIAN women had approximately twice the odds of acute alcohol intoxication relative to White women at the time of death.^30^ Also, increased alcohol use is known to be associated with mortality among people with HIV.^31^ This risk disproportionately affects Black women, whose incidence rate for HIV far exceeds that of White women (estimated at 783.7 and 43.6 per 100,000 for Black and White women, respectively).^32^

Research also indicates socioeconomic differentials in alcohol-related morbidity and mortality. An English study of hospital admissions from 2010 to 2013 that examined wholly and partially alcohol-attributable conditions found the greatest socioeconomic disparities among women with wholly alcohol-attributable chronic and acute conditions.^33^ These results suggest that socioeconomic status differences in harmful drinking patterns contribute to differential morbidity.

Applying a similar comparative approach, Probst and colleagues conducted a meta-analysis of 15 studies from 7 countries and found greater socioeconomic disparities in women’s alcohol-attributable mortality than in their all-cause mortality.^34^ Across different measures of socioeconomic status (e.g., individual-level education, occupation, employment status, or income), socioeconomically disadvantaged women had 1.8 times the relative risk of alcohol-attributable vs. all-cause mortality when compared to more advantaged women. Similarly, a Scottish study of women and men combined found that socioeconomically disadvantaged participants who drink moderately had much greater risk for alcohol-attributable harms (i.e., hospital admissions or deaths) compared to socioeconomically advantaged participants who drink moderately or even heavily, regardless of the socioeconomic status measure used and even after controlling for differences in binge drinking, obesity, smoking, and other risk factors.^20^

Other research has investigated disparities in the protective health effects of moderate drinking. Although protective effects for cardiovascular disease mortality and for diabetes onset have been found,^35^,^36^ some studies indicate health benefits for Whites but not for racial/ethnic minorities.^37^–^39^ Race/ethnicity differences in the protective effects of alcohol have also been observed in two studies of all-cause mortality. One study used NAS data^40^ and the other was a gender-stratified study based on data from the National Health Interview Survey.^41^ The latter study found that moderate drinking was associated with the lowest mortality among White women (a mortality rate of 40.1 per 1,000 person-years). In Black women, moderate drinking was associated with a mortality rate of 93.8 per 1,000 person-years), more than double the rate of White women with a similar drinking level and also higher than the mortality rate associated with high-risk drinking among Black women (67.6 per 1,000 person-years), although confidence intervals for Black women’s rates were widely overlapping.^41^

In contrast to these disparities, the United States has seen a racial/ethnic crossover in liver cirrhosis mortality rates for women. Although rates for Black women were highest in 2000, they have since dropped, and rates for White, non-Latina women and for White, Latina women have risen, exceeding the rates for Black women.^42^ These results are consistent with reports of increased consumption and alcohol problems among White women based on the 2000 and 2010 NAS survey series.^14^,^43^

## POSSIBLE EXPLANATIONS FOR DISPARITIES

An obvious potential explanation for these disparities is that they reflect population differences in harmful drinking patterns. Sexual minority women, for instance, are more likely to drink alcohol, to drink to intoxication, and to drink heavily compared to exclusively heterosexual women (adjusted *OR* = 1.8 and 2.0 for intoxication and heavy drinking, respectively).^27^ Yet, it is unlikely that consumption patterns alone account for disparities. Indeed, the finding of greater harm despite lower or similar levels of drinking lies at the heart of the alcohol harm paradox. As noted, the latter refers to socioeconomic disparities in alcohol outcomes but is similar to the phenomenon observed for some racial/ethnic minority groups of disparities in alcohol problems at the same level of heavy drinking among both women and men. Related to this, it is important to note that previous research finding elevated alcohol consumption among AIAN relative to White individuals has been based on specific AIAN tribes or geographic-area subgroups, whose prevalence of alcohol use varies.^44^ Recent analyses of the 2009 to 2013 NSDUH and the 2011 to 2013 Behavioral Risk Factor Surveillance System indicate that, nationally, AIAN and White participants had similar odds of binge drinking and heavy drinking (i.e., drinking five or more drinks on 5 or more days). Moreover, White participants had lower abstinence relative to AIAN participants, with an adjusted odds ratio for abstinence among White participants relative to AIAN participants of 0.64 (95% CI: 0.56, 0.73).^45^

Thus, consideration of other ways that disparities in alcohol-related problems can arise is needed. Recent research calls attention to potential explanations involving the life course, differential vulnerability, and access to care. As noted earlier, this review reflects a predominant focus in the literature on racial/ethnic and socioeconomic disparities. Future studies are needed to assess relevance to other disadvantaged social groups.

### Harmful Drinking Patterns Over the Life Course

Reflecting core concepts of life-course developmental theory,^46^ both the age at which heavy drinking occurs and the duration of heavy drinking across the life course are relevant to disparities in alcohol-related problems. This makes sense intuitively, as the longer a person engages in health risk behaviors, the greater the chances of experiencing related problems. Also, certain age periods are likely to pose more or less risk for different kinds of alcohol-related problems. Bouts of heavy drinking, for instance, are likely to be tolerated less and to have more consequences when coupled with greater responsibilities to others, such as family and employers.

Notably, three recent studies based on National Longitudinal Study of Adolescent to Adult Health data examined racial/ethnic differences in the heavy-drinking trajectories of young women, with somewhat mixed results (possibly reflecting methodological differences, such as adjustments for socioeconomic status).^47^–^49^ Two studies showed that heavy drinking of young White women consistently exceeded that of Black women.^47^,^48^ One study indicated that the rapidly declining trajectory of White women converged with the trajectory of Latina women by age 30,^47^ and another showed a convergence of White, Latina, and Black women’s trajectories by their early 30s.^49^

A fourth study based on the 1979 cohort of the National Longitudinal Study of Youth (NLSY) examined women’s heavy-drinking trajectories from ages 21 to 51.^50^ This study also found that heavy drinking among White women exceeded that of Black and Latina women in their early and mid-20s, but the trajectories of all 3 groups declined thereafter, with no significant racial/ethnic differences in heavy drinking between ages 30 to 51. However, sensitivity analyses excluding lifetime abstainers and women who never drank heavily showed a crossover in the heavy-drinking trajectories of Black and White women.^50^ The trajectory for Black women rose during their early 20s, a period when White women’s trajectory declined, thus causing a crossover at age 30. Thereafter, Black women’s trajectory declined and reconverged with the flattening trajectory for White women at age 40. Consistent with these results, a 2010 NAS analysis of heavy drinking trajectories among women who reported ever drinking in their lifetime found that Black women, compared to White women, had twofold greater odds of persistent, frequent, heavy drinking (vs. declining heavy drinking) beyond their 20s and into their 40s (adjusted *OR* = 2.65, *p* < .01).^51^

Taken together, these life-course drinking studies highlight racial/ethnic differences in the heavy-drinking trajectories of women in their early and mid-20s, which are consistent with the greater DSM-IV AUD risk observed during this period among young White women. Importantly, early adulthood is a time when health is relatively robust, and many women have yet to take on large, adult responsibilities. Drinking trajectory studies that extend beyond the 20s are rare, but there is some evidence of Black–White disparities in the age and duration of heavy drinking among women who reported ever drinking in their lifetime. These disparities were found for women in their 30s, possibly extending to their 40s.

Prospective studies beyond young adulthood are needed, especially for younger cohorts, as racial/ethnic differences in heavy drinking may be changing.^1^,^52^ Nonetheless, the observed Black–White disparity in heavy drinking after young adulthood is consistent with the findings from a NESARC study of women who drink (described earlier), showing greater DSM-IV AUD onset among Black women in their 30s and 40s, as well as greater AUD persistence among Black women in their 40s and older, compared to White women in these same age groups as well as younger (ages 18 to 29).^16^ These disparities are particularly significant when juxtaposed with other life-course findings. Namely, by midlife, there are striking racial differences in cumulative lifetime exposure to socioeconomic disadvantage,^53^ and disparities in health become more pronounced.^5^,^54^

### Cumulative Disadvantage

Population differences in exposure to health risk factors and their cumulative effects are an important mechanism in health disparities.^5^ Cumulative disadvantage refers to the notion that social status positions such as race/ethnicity and socioeconomic status profoundly influence opportunities and resources over the life course and, thus, also affect exposures to health risk factors.^55^

Growing up in poverty in neighborhoods with inferior schools, greater crime and violence, and limited economic opportunities can lead to poor quality and low-paying jobs, a lack of health insurance, and ongoing exposure to stressors. Black women and men with low incomes are particularly affected by these factors due, in part, to racial residential segregation^56^ and geographic inequalities of opportunity.^57^ Consistent with this, research has indicated that a large majority of Black children who were raised in poor neighborhoods continue to reside in similar neighborhoods as adults.^58^

In an early articulation of the effects of cumulative disadvantage and its relationship to health disparities, Geronimus proposed the “weathering hypothesis” to account for the accelerated health deterioration of Black persons relative to White persons.^59^ This is exemplified by high rates of chronic disease found in young and middle-aged Black women residing in low-income, urban areas, which contribute to their early mortality rates. According to the hypothesis, the widening racial health disparity seen through middle adulthood reflects the cumulative effect of adverse exposures from conception onward. These adverse exposures include chronic social stressors (e.g., discrimination), environmental hazards, inadequate health care access and treatment, and unhealthy behaviors. Notably, greater alcohol availability, targeted advertising, and less access to healthy food in low-income and minority neighborhoods can contribute to and aggravate unhealthy behaviors.^60^–^62^

Research has since shown that chronic, enduring stress affects the body’s physiological stress response, with adverse effects on the cardiovascular, metabolic, and immune systems.^63^ Moreover, the physiological consequences of chronic stress, which are referred to as allostatic load and assessed via biomarkers, have been found to be greater among poor and non-poor Black women than White women, and have been associated with accelerated aging.^64^,^65^ Consistent with these findings, data from the 2017 National Health Interview Survey showed that 14% of Black women (and 13% of Latina women) reported fair or poor health, in contrast to 8% of White women.^66^ Even when the sample was stratified by poverty status (i.e., poor, near poor, and not poor, with poor defined as having income below the federal poverty threshold), Black women and men tended to report worse health than White women and men.

As suggested, cumulative disadvantage can also affect health indirectly through risky health behaviors that people use to cope with stressors.^67^ A longitudinal study based on NESARC data found that the effect of poverty on heavy drinking incidence was worse for Black women who drink than for their Latina and White counterparts.^68^ A different longitudinal study based on the 1979 NLSY cohort data reported that cumulative poverty across the life span was positively associated with onset and persistence of alcohol dependence symptoms after young adulthood (in a combined sample of women and men who drink).^69^ Further, a study based on 2010 NAS data found that cumulative socioeconomic disadvantage partly explained the disparity in persistent heavy drinking until midlife between Black and White women.^51^

This confluence of disparities in cumulative disadvantage and health in middle adulthood provides an important backdrop for understanding disparities in alcohol problems after young adulthood. It raises the question of differential health vulnerability—the idea that certain social groups are more susceptible to health-related consequences when they are exposed to risk factors such as, in this case, heavy drinking.^70^ To the extent that health “weathering” begins to accelerate after young adulthood and at a faster rate for demographic groups that have more enduring chronic stress, heavy drinking beyond young adulthood may contribute to alcohol-related health disparities at midlife and later. In keeping with this, a recent NLSY study by Kerr and colleagues found that among Black and Latina women, but not White women, diabetes onset was associated with a history of heavy drinking in the previous 10 years, even when controlling for health risk behaviors, socioeconomic status, and other demographics.^71^

Differential health vulnerability may reflect various mechanisms that require future study. It may be rooted in biological interactions with alcohol that affect health. For example, heavy drinking can exacerbate certain health conditions such as hypertension, type 2 diabetes, and chronic kidney disease, which are more prevalent among Black Americans. Also, as discussed by Jackson and colleagues, differential vulnerability may reflect unmeasured health risk behaviors like smoking and unhealthy eating, which may co-occur with heavy drinking and are thus potentially confounding variables.^41^

Alternatively, unhealthy behaviors could, in some instances, be effect modifiers that interact with alcohol to alter risk for health conditions. For instance, the aforementioned NLSY study by Kerr and colleagues found an interaction between alcohol and obesity for diabetes risk for women.^71^ Bensley and colleagues’ study of male, Veterans Health Administration patients who had HIV provides further illustration of this complexity.^31^ Black patients with low-risk drinking (defined as a score of one to three on the Alcohol Use Disorders Identification Test consumption questions [AUDIT-C]) had greater mortality than White patients who had similar drinking levels, indicating differential vulnerability. The disparity was attenuated after adjusting for the greater presence of hypertension, hepatitis C, tobacco use, and other drug use among Black patients. To better understand alcohol-related disparities and the epidemiologic paradox of greater problems despite lower levels of drinking for some groups, research is needed to examine population differences in health and health behaviors and potential interactions with alcohol consumption patterns.

### Other Social and Biological Factors

Studies have documented gene variants that are more prevalent among Black persons^21^ that affect the metabolism of alcohol, leading to a buildup of acetaldehyde in the bloodstream. While the gene variants have been associated with lower rates of alcohol dependence and heavy drinking, experimental research by Pedersen and McCarthy has found that the variants also are associated with more intense subjective responses to alcohol.^72^ Specifically, they found that Black participants experience greater stimulating effects from alcohol than White participants, even after controlling for differences in past-month alcohol use. Further, greater increases in stimulation are associated with more alcohol-related problems among Black participants. As the researchers suggested, this acute stimulation could contribute to disparities in the negative consequences of drinking alcohol at a given level of consumption.^72^

In addition, Black women in this study experienced greater sedating effects from alcohol than White women. In view of the greater cumulative and chronic stress experienced by Black women compared with White women,^51^,^65^ this finding of greater sedating effects of alcohol might be a factor in Black-White disparities in persistent heavy drinking and AUD among older women who drink.

Social position and sociocultural context also affect the likelihood of experiencing alcohol problems, particularly negative social consequences, at a given level of consumption. For years, researchers have called attention to the greater negative consequences of drinking borne by racial/ethnic minority groups who have less permissive drinking norms and are subject to greater societal scrutiny and stigmatization.^73^,^74^ People with greater resources and higher status are better able to shield themselves from the negative consequences of drinking that others experience.^75^ For example, negative consequences could be minimized at work (because of greater flexibility and autonomy and less scrutiny), in family duties (by paying for childcare or home-delivered meals and groceries), and when going out for the night (by hiring a driver).

These differential standards and consequences of drinking may be seen among women, perhaps more now than in the past when gendered roles and drinking norms were more similar across women. Reflecting on recent decades, Schmidt observed that social and economic changes resulting in greater freedoms for women have led to the “equal right to drink” only for women in the middle and upper classes.^76^ By contrast, women with low incomes and women who receive welfare benefits, particularly racial/ethnic minority women, arguably have been more surveilled, stigmatized, and penalized for alcohol and other drug use.

Finally, stress experienced due to being a member of a stigmatized minority group may help to explain alcohol-related disparities between sexual minority women and exclusively heterosexual women. Minority stress theory applied to drinking behavior suggests that the heavy drinking patterns of sexual minority women (relative to heterosexual women) are related to the stress of holding one or more minority identities.^77^,^78^

Minority stress theory has been used in many studies. Research shows that sexual minority women experience stressors such as discrimination and harassment because of their sexual orientation, and that these women are more likely to report psychological distress than heterosexual women.^74^ A study of sexual minority women and sexual minority stressors associated with substance use and mental health outcomes (e.g., unfair treatment, events of prejudice, and victimization) has provided further empirical support of this theory.^79^ In this study, sexual minority stressors mediated the adverse effects of more masculine gender expression (i.e., a set of culturally assigned qualities to the category of masculine) on mental health and substance use outcomes. Other studies have found that sexual minority women experience additional stressors associated with increased alcohol use. In comparison to exclusively heterosexual women, sexual minority women are more likely to have experienced child sexual abuse, depression in their lifetime or in the past 12 months, and early onset of alcohol use.^26^,^80^

Together, this varied literature suggests that social and biological factors may contribute to alcohol-related disparities among women in several ways. These factors may increase exposure to high levels of stress and discrimination (and drinking in response), they may increase sensitivity to the physiological effects of alcohol, and they may increase exposure to punitive societal responses to an individual’s own alcohol use.

### Differential Access to and Quality of Care

Differences in access to care and in the quality of care received constitute another important explanation for disparities in alcohol-related problems. Although health care access and quality account for a relatively small percentage of the variation in life expectancy in the United States—estimated at 10%^81^—health care is a valuable resource. Indeed, having a regular source of primary care has been associated with reduced racial/ethnic and socioeconomic disparities in health.^54^

The Institute of Medicine’s report, *Unequal Treatment*, famously documented racial/ethnic disparities in the quality of health care received in the United States, even after accounting for differences in socioeconomic status, insurance, disease stage, comorbidities, and facility type.^82^ Such findings have motivated the national goal of ensuring equitable access to high-quality care to mitigate disparities in early or delayed diagnosis, types of treatment, and care outcomes.^83^ Part of the problem of health care disparities is structural, related to income, insurance, and the type and quality of care that is affordable and geographically accessible. Another part of the problem is social, related to implicit (unconscious) bias on the part of health care providers and how this bias affects patient-provider communication and interaction, treatment decisions, and health care outcomes.^84^,^85^ Related to both structural and social factors, health care utilization also reflects patient perceptions, attitudes, and willingness to seek care. In the case of racial/ethnic disparities in alcohol-related care or treatment, cultural acceptability (including language compatibility) and perceived stigma toward people with AUD may be particularly relevant.^86^,^87^

Whereas considerable research has investigated racial/ethnic and gender disparities in the receipt of alcohol-related care, far less is known about disparities among women specifically. In a rare, gender-stratified analysis of alcohol treatment utilization, Zemore and colleagues’ analysis of NAS data found racial/ethnic disparities in treatment use among women with a lifetime AUD.^88^ When compared with White women, Latina and Black women were significantly less likely to obtain specialty alcohol treatment, even after controlling for survey year, age, socioeconomic status (i.e., education and income), and insurance status (adjusted *OR* = 0.31 and 0.38 among Latina and Black women, respectively; *p* < .05). Moreover, this disparity was also observed for Alcoholics Anonymous use (adjusted *OR* = 0.38 and 0.37 for Latina and Black women, respectively).^88^ Other studies (using samples of women and men combined) have further shown disparities in treatment completion, which is an important predictor of post-treatment substance use and health outcomes.^89^,^90^

A variety of factors might contribute to racial/ethnic disparities in treatment use specifically among women. One factor is the stigma of AUD, which may be a particularly salient deterrent for social groups that have more conservative drinking norms and that might already be socially marginalized. Notably, there is evidence of more conservative drinking norms for Black women compared to those for White women^91^ and less permissive attitudes toward Latina women’s drinking, which tend to be held by less-acculturated Latina women.^92^ The stigma of AUD could lead to concealment or denial of alcohol problems and to family concerns about privacy and pressure to not seek treatment. All of these issues may be magnified for women due to the more intense social control of women’s drinking.

Other potential treatment barriers are a lack of childcare and concerns that children could be taken away. These concerns are not unfounded, given research showing that Black mothers who use alcohol or other drugs are reported to child protective services more often than similar White mothers.^93^ In addition, women generally are more likely than men to experience treatment barriers because of transportation difficulties and inadequate insurance.^94^ The latter may be particularly relevant to racial/ethnic minority women, as studies have found that Latinx and Black individuals are more likely than White individuals to report logistical and structural barriers.^95^,^96^ Considering the pronounced racial/ethnic disparities in alcohol problems among women after young adulthood, additional disparities in alcohol-related care and treatment compound the problem. This large unmet need among minority women, which may reflect a variety of causes, must be addressed.

## CONCLUSION

This review provides evidence of alcohol-related disparities among women. The research in this area is relatively sparse, but disparities in AUD prevalence, the negative consequences of drinking, and alcohol-related health, morbidity, and mortality outcomes are apparent. This review also highlights the importance of a life-course perspective for understanding disparities in alcohol problems. By examining what happens within and between social groups across the life span, the widening of social group differences in cumulative socioeconomic disadvantage, health, and alcohol-related problems—especially after young adulthood—becomes more noticeable. Future research is needed to examine how these various disparities may be interrelated.

Importantly, a life-course lens also requires attending to social roles and health as these change with age. Attention to such changes can help to advance understanding of how alcohol consumption results in negative consequences and why some groups are affected more than others. Finally, social position and sociocultural context remain important considerations because they can affect internal and external responses to drinking. Social position and sociocultural context also influence access to, use of, and the quality of alcohol-related and general health care. All these factors can affect the persistence of alcohol-related problems and the progression of disease.

In thinking about potential remedies, education emerges as one important factor. Some research has found that education, compared with income, is more strongly and negatively associated with the onset of disease (i.e., the likelihood that an individual will develop a chronic health condition). By contrast, income is a stronger predictor than education of how a disease progresses once an individual has the condition.^97^ In light of the benefits of education for health and health behavior,^50^,^98^ improving access to quality education at an early age and supporting higher educational attainment is an important strategy for improving health and addressing health disparities among racial/ethnic minorities and socioeconomically disadvantaged persons.

In addition, increasing insurance coverage and access to affordable, quality health care for underserved groups, a goal of the Patient Protection and Affordable Care Act, represents another crucial path to reducing health disparities. However, efforts devoted to improving health care access and quality will yield limited gains so long as stress and social stigmatization among minority populations persist, and profound differences in neighborhood conditions and available opportunities remain. These are the fundamental causes that need to be addressed to truly eliminate alcohol-related and general health disparities.

## Figures and Tables

**Table 1:** 2017 NSDUH 12-Month Prevalence of DSM-IV Alcohol Dependence and AUD Among Women

	Alcohol Dependence, % (Standard Error)		Alcohol Dependence or Abuse, % (Standard Error)	
Category	All Women (*N* = 22,567)	Drank in Past Year (*N* = 16,042)	All Women (*N* = 22,567)	Drank in Past Year (*N* = 16,042)
**Race/Ethnicity**				
**White** †	2.70 (0.14)	3.70 (0.20)	4.44 (0.15)	6.07 (0.22)
**Black**	1.86 (0.24)*	3.11 (0.41)	3.12 (0.31)**	5.21 (0.50)
**AIAN**	8.04 (1.26)**	16.21 (2.64)**	9.10 (1.32)**	18.35 (2.75)**
**Native Hawaiian/Pacific Islander**	2.11 (1.54)	4.46 (3.27)	2.90 (1.71)	6.11 (3.62)
**Asian**	1.29 (0.42)*	2.68 (0.85)	1.79 (0.46)**	3.71 (0.88)*
**More Than One Race**	4.91 (1.70)	7.44 (2.63)	6.70 (1.76)	10.15 (2.75)
**Latina**	1.72 (0.23)**	2.93 (0.42)	3.20 (0.28)**	5.46 (0.52)
**Education**				
**Less Than High School**	1.58 (0.24)**	3.92 (0.61)	2.11 (0.32)**	5.24 (0.79)
**High School Graduate**	1.60 (0.15)**	2.80 (0.27)	2.63 (0.19)**	4.61 (0.34)*
**Some College**	3.05 (0.27)	4.23 (0.39)	4.84 (0.32)	6.72 (0.45)
**College Graduate** †	2.69 (0.22)	3.38 (0.27)	4.74 (0.27)	5.96 (0.33)
**Sexual Identity**				
**Heterosexual** †	2.14 (0.11)	3.18 (0.17)	3.61 (0.12)	5.36 (0.19)
**Lesbian**	5.12 (1.33)**	6.31 (1.62)*	8.21 (1.69)*	10.12 (2.10)**
**Bisexual**	8.63 (1.02)**	10.68 (1.25)**	12.23 (1.11)**	15.12 (1.35)**

*Note:* Data are for women ages 18 and older. Percentages are weighted for sampling, and sample size (*N*) represents unweighted totals. Pairwise significance tests involve comparisons to the reference category using Pearson’s chi-square test.

* *p* < 0.05,

** *p* < 0.01,

† = reference category.

*Source:* Data from Substance Abuse and Mental Health Services Administration, Center for Behavioral Health Statistics and Quality, October 2018.^15^
